# Comprehensive Approach of a Traumatized and Discolored Anterior Tooth in Geriatric

**DOI:** 10.1155/crid/9959232

**Published:** 2025-12-29

**Authors:** Diani Prisinda, Kartika Tria Sulendra, Dudi Aripin

**Affiliations:** ^1^ Department of Conservative Dentistry, Universitas Padjadjaran Fakultas Kedokteran Gigi, Bandung, West Java, Indonesia

**Keywords:** case report, dental trauma, elderly endodontics, external root resorption, internal bleaching

## Abstract

The prevalence of dental trauma, particularly in permanent maxillary anterior teeth, is high and can significantly impact a patient′s quality of life. Furthermore, the growing desire of elderly patients to maintain their teeth has led to an increased need for the performance of complex dental treatments. Traumatic injuries often lead to root resorption, pulp necrosis, as well as wide root canals and open apices due to disruption of tooth development. Therefore, precise strategies are required for working length determination, cleaning, and shaping, along with obturation. A 64‐year‐old female patient came with a chief complaint of a gradually darkening front upper tooth. The patient has a history of traumatic injury where she fell and hit her upper left central incisor. The tooth is clinically darkened, and radiographic examination revealed a periapical lesion, wide root canal, and slightly convergent apex as Stage IV Cvek classification as well as external root resorption. Endodontic treatment was done on Tooth 21 using a hybrid technique with a combination of brushing and balanced‐force motion in the cleaning and shaping procedure. The treatment was completed with warm vertical compaction obturation and followed by internal and external bleaching as well as direct restoration. Endodontic treatment utilizing a hybrid cleaning and shaping technique, combined with bioceramic sealer and warm vertical compaction obturation, is effective in posttraumatized elderly teeth resulting in the healing of periapical lesions. Internal bleaching successfully addressed the coronal discoloration followed by direct composite restoration achieving a favorable aesthetic outcome.

## 1. Introduction

Trauma to the oral and maxillofacial region is common and comprises 5% of all injuries [1]. While traumatic injuries to immature teeth are well recognized for their risk of pulp necrosis and compromised root development in young patients [2, 3], their occurrence and management in elderly patients remain less frequently documented. In older individuals, traumatic pulp necrosis may go undetected, particularly in the absence of microbial contamination, making diagnosis challenging. Moreover, misconceptions about the poor prognosis of root canal therapy in elderly patients (often attributed to technical difficulties, systemic limitations, or lack of motivation to preserve natural dentition) can further delay appropriate treatment [4–6].

External inflammatory resorption (EIR) represents one of the most destructive sequelae of dental trauma, arising from root surface injury and subsequent osteoclastic activity [7]. In teeth with wide canals and thin dentinal walls, endodontic management is particularly complex due to the risk of root fracture, difficulties in instrumentation, and obturation challenges. Achieving effective chemomechanical disinfection and obtaining a three‐dimensional fluid‐tight seal combined with bioceramic sealer (high biocompatibility and low cytotoxicity and viscosity) remain critical to halting resorption and preserving tooth structure [8–12].

Beyond biological preservation, traumatized teeth may exhibit various unaesthetic discolorations as a result of different dental trauma sequelae. Posttraumatic discoloration results from hemolysis byproducts and their penetration into dentinal tubules, leading to darkening of the crown [13]. While intracoronal bleaching is widely accepted as a conservative and effective aesthetic solution, few reports have combined it with external bleaching to enhance the outcome in traumatized, endodontically treated teeth [14, 15].

Limited literature describes successful endodontic management of an elderly patient presenting with a wide canal and EIR following trauma, followed by a combined internal–external bleaching protocol and direct composite restoration for aesthetic rehabilitation. This report highlights such a case, emphasizing the integration of advanced hybrid instrumentation, warm vertical compaction, bioceramic sealer obturation, and staged bleaching to restore both function and aesthetics.

## 2. Case Report

A 64‐year‐old female patient came to RSGM Universitas Padjadjaran with a chief complaint of a gradually darkening front upper tooth. The patient has a history of traumatic injury where she fell and hit her upper left central incisor when she was 9 years old. Then, she suffered trauma again 20 years later, which caused tooth mobility for several days. Now, she has no symptoms of tenderness or swelling in the area. Medically, no other systemic complications were declared or detected. She accepted all the treatments explained and wanted the tooth to be lightened again.

Clinical examination showed a discolored upper left central incisor (Figure 1), and there is no response in the thermal test, electric pulp test, percussion, and biting test. Radiographic examination revealed a wide root canal and shortening of the tooth with a nonblunderbuss apex. The radiographic image also showed widening of the periodontal ligament and lamina dura as well as external resorption characterized by a ragged bowl‐shaped indentation along the lateral border of the root surface with an adjacent periradicular radiolucency (Figure 2a). Based on these findings, it was determined that a diagnosis of pulp necrosis with asymptomatic apical periodontitis was made. An immature tooth presented as Stage IV in the Cvek classification with EIR. The diagnosis and extent of pathology can be sufficiently assessed through conventional periapical radiographs due to the large and clearly visible wide canal, open apex, and resorptive defects. Radiation dose optimization, the cost‐effectiveness, and the accuracy of the apical gauging technique suggest that CBCT imaging may not be justified.

Figure 1Intraoral preoperative photograph of discolored Tooth 21. (a) Labial view matched ND‐8 shade guide. (b) Palatal view.

**Figure:**
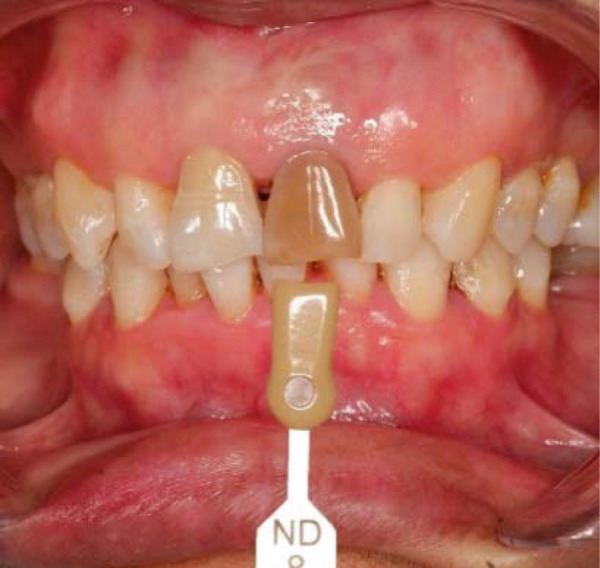
(a)

**Figure:**
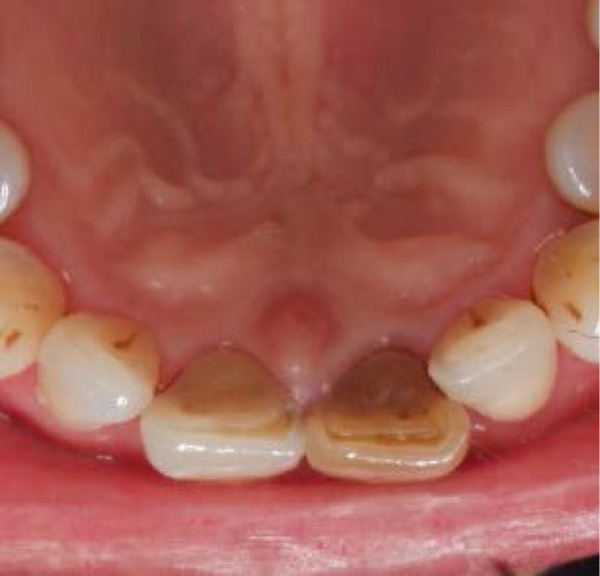
(b)

Figure 2(a) Periapical radiograph showing immature Tooth 21 with external resorption (yellow arrows) and periradicular radiolucency. (b) Working length and apical gauging confirmation.

**Figure:**
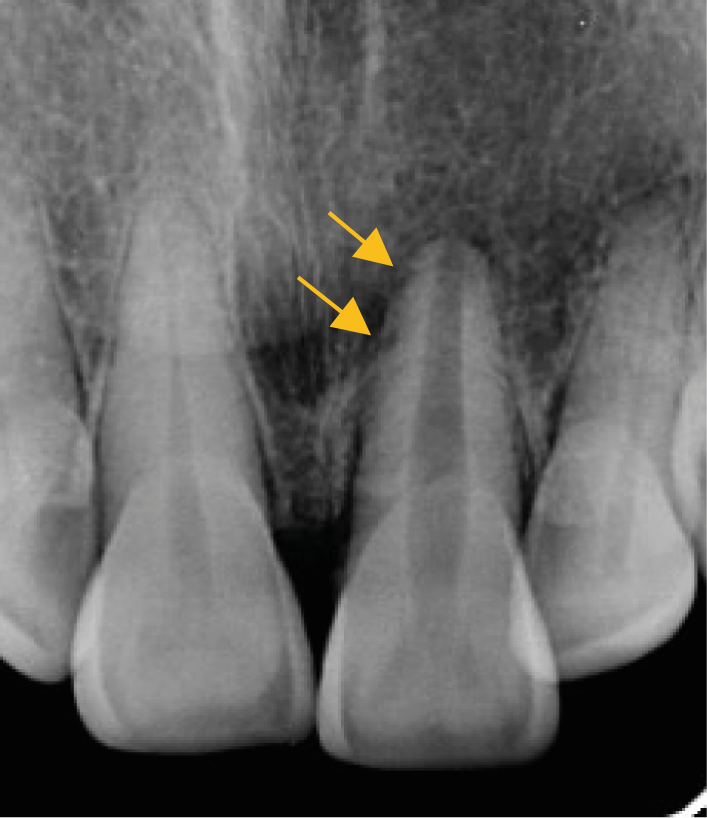
(a)

**Figure:**
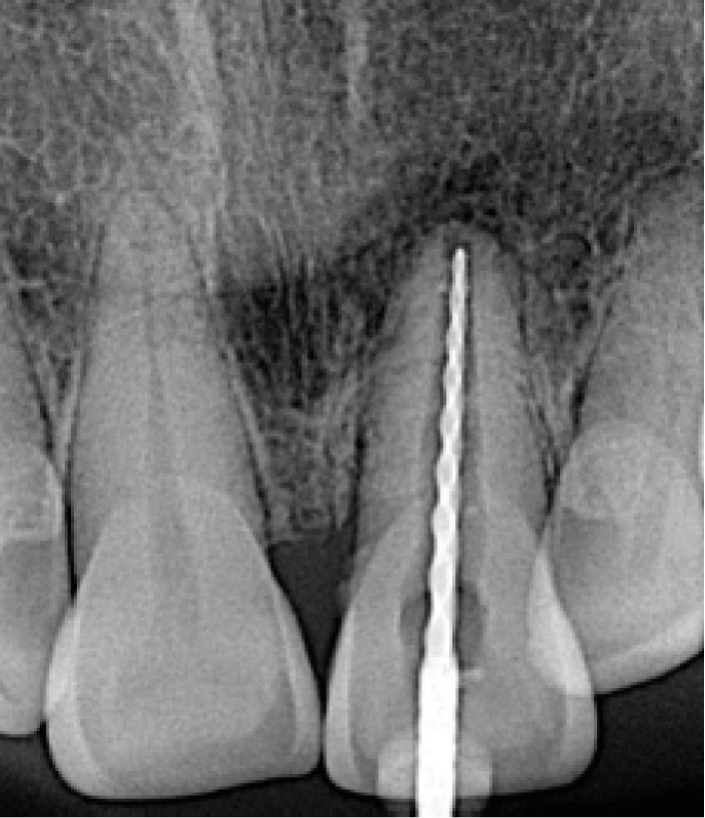
(b)

A treatment plan was developed that included root canal treatment and internal bleaching. Informed consent was obtained, dental health education was provided, and scaling was done prior to root canal treatment. All of the procedures, including carious removal, access cavity preparation, and the remainder of the root canal treatment, were carried out using rubber dam isolation. After scouting the root canal with a #10K‐File M‐Access (Dentsply Maillefer, Ballaigues, Switzerland) to ensure patency, the coronal flaring was done using SX ProTaper Gold (Dentsply Sirona, Ballaigues, Switzerland). Working length was determined using an apex locator (Root ZX II, J. Morita Corp., Kyoto, Japan), and apical gauging was confirmed with a periapical radiograph (Figure 2b). Preparation of a wide canal and slightly convergent apex used the hybrid technique. This technique combined the brushing motion of shaper files using S1‐S2 ProTaper Gold (Dentsply Sirona, Ballaigues, Switzerland), which has a variable progressive taper, and balanced force motion using #25–#60K‐File M‐Access. The file was inserted and then rotated a quarter turn clockwise while applying light apical pressure. Then, the file was rotated three‐quarters turn in a counterclockwise direction with sufficient apical pressure to hold the instrument at the proper working length. Then, the file was rotated a quarter turn clockwise while being pulled out.

During the procedure, all file tips were cleaned from debris to prevent debris packing and loss of working length. The patency was maintained in the root canal prior to and during the chemomechanical cleaning. The canal was then irrigated with 2.5% sodium hypochlorite and activated using an ultrasonic file. A 17% EDTA solution and normal saline were also used for the final rinse, and then a dressing of calcium hydroxide was given in the canal. Calcium hydroxide was applied as an intracanal medicament periodically every 2 weeks because its effectiveness loses overtime and must be replaced to maintain its therapeutic properties. After two changes of the medicament, the patient reported no complaints, there was no pain on percussion or palpation, the root canal was dry, the resorptive lesion had ceased, a uniform periodontal ligament space had formed, periapical healing was observed, and no new radiolucent areas were visible.

At the next appointment, a 5% tapered gutta‐percha (Dentsply Sirona, Ballaigues, Switzerland) was trimmed to fit at the working length. A periapical radiograph revealed a decrease in the radiolucency and gutta‐percha fit at the apex at a diameter of 0.6 mm (Figure 3a). The canal and master cone were lightly coated with bioceramic sealer (CeraSeal, Meta Biomed Co. Ltd., Cheongju, Korea) before being placed into the canal, and a warm vertical compaction technique was used to fill the root canal (Figure 3b).

Figure 3(a) Master cone fitting and gutta‐percha trial radiograph. (b) Postobturation periapical radiograph showing hermetic obturation.

**Figure:**
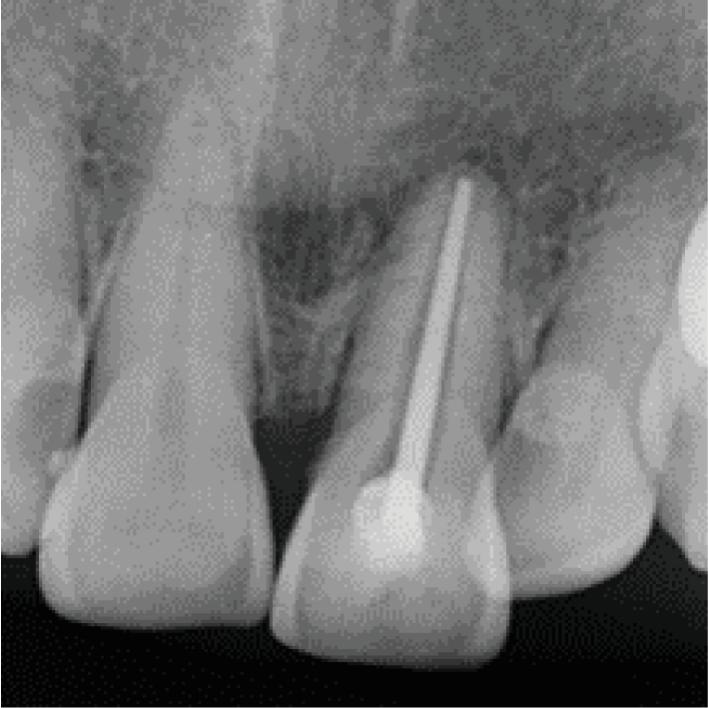
(a)

**Figure:**
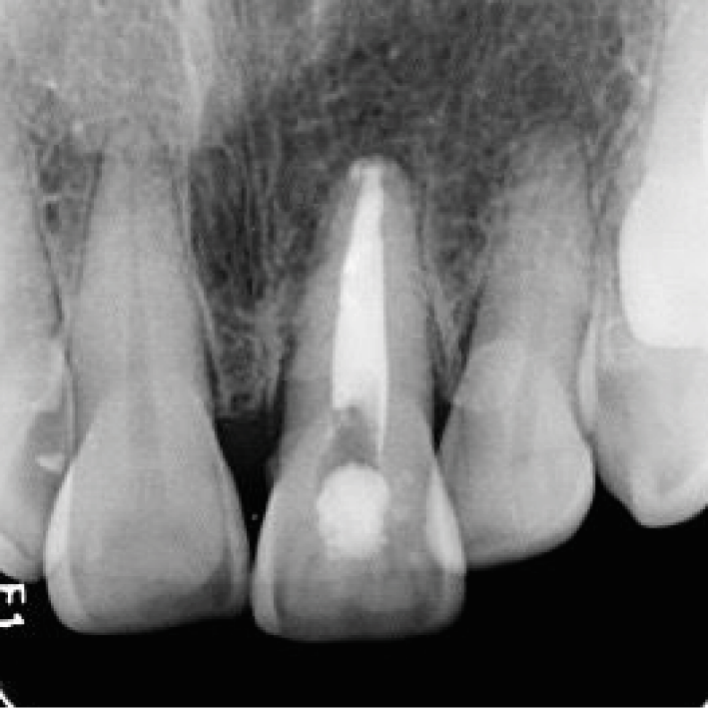
(b)

The next visit to begin the bleaching treatment, an examination was taken to assess the conditions of the endodontic treatment and periodontal tissue. There was no periapical lesion, and endodontic treatment was satisfactory. The tooth color was matched using the ND shade guide (Ivoclar Vivadent, Schaan, Liechtenstein). In this case, the initial color was ND‐8. After isolating the teeth with a rubber dam, root canal filling was removed 2 mm below the cementoenamel junction, and then the cavity was washed with normal saline and dried. To prevent leakage in the coronary region, 2 mm thick resin modified glass ionomer cement (RM‐GIC) GC Fuji II LC (GC Corporation, Tokyo, Japan) was placed in the orifice of the root canal (Figure 4a). The resultant shape from the facial view is the “bobsled tunnel” outline, and from the proximal view, it resembles a “ski‐slope.” The cavity was irrigated with hypochloride and saline to remove the smear layer and debris. Hydrogen peroxide 35% (Opalescence Endo, Ultradent, Utah, United States) was placed in the pulp chamber, a layer of polytetrafluoroethylene (PTFE) was tightly placed over this, and the access cavity was sealed with glass ionomer cement GC Fuji IX (Figure 4b,c).

Figure 4Internal bleaching procedure. (a) Barrier placement using RM‐GIC, (b) application of hydrogen peroxide 35%, and (c) temporary restoration.

**Figure:**
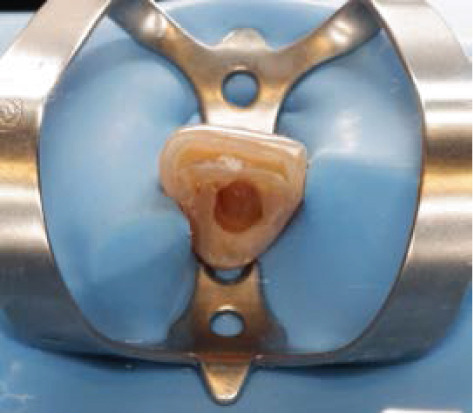
(a)

**Figure:**
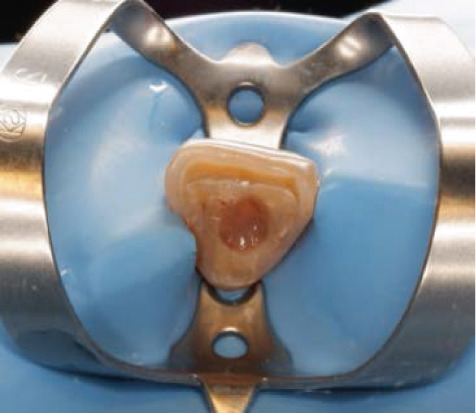
(b)

**Figure:**
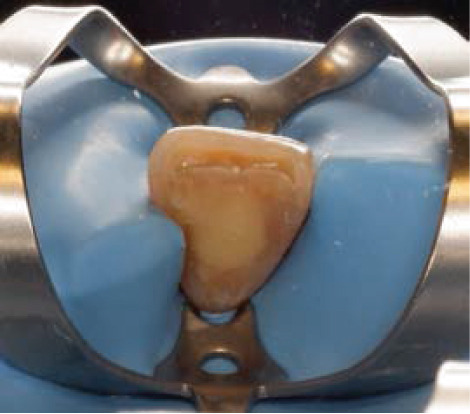
(c)

The bleaching agent was replaced three times until obtaining the desired color (Figure 5), with intervals of 5 days between each session and the tooth color recorded at ND‐2 shade. Subsequently, the cavity was washed thoroughly and filled with a temporary restoration.

Figure 5Shade comparison. (a) Initial condition, (b) first internal bleaching, (c) second session, and (d) final result.

**Figure:**
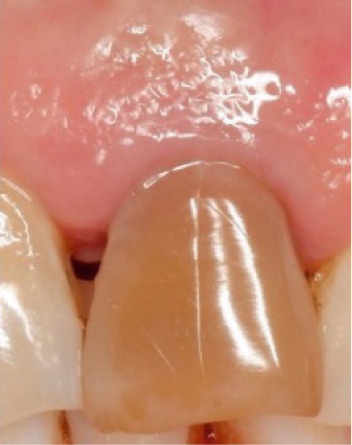
(a)

**Figure:**
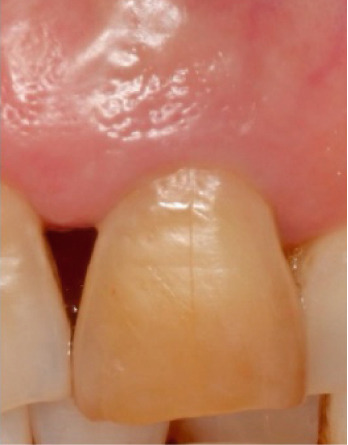
(b)

**Figure:**
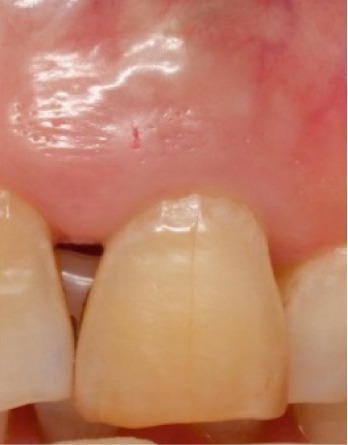
(c)

**Figure:**
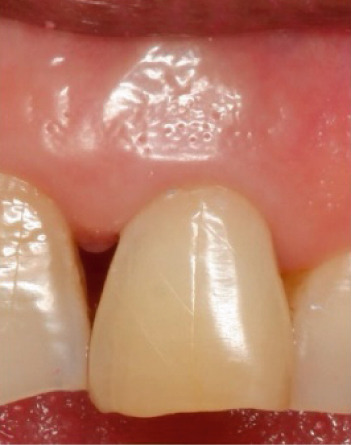
(d)

After 1 week, the tooth underwent complementation with external bleaching to correct the minimal persistent color change even after the completion of internal bleaching. This procedure involves gingival isolation with OpalDam, which was light cured for 20 s and protects the adjacent teeth with PTFE. Then, the gel of Opalescence Boost PF 40% (Ultradent, Utah, United States) was applied for 15 min, and this procedure was performed three times in one session (Figure 6).

Figure 6External bleaching procedure. (a) Final shade of internal bleaching, (b) application of hydrogen peroxide 40% gel, and (c) final shade of external bleaching.

**Figure:**
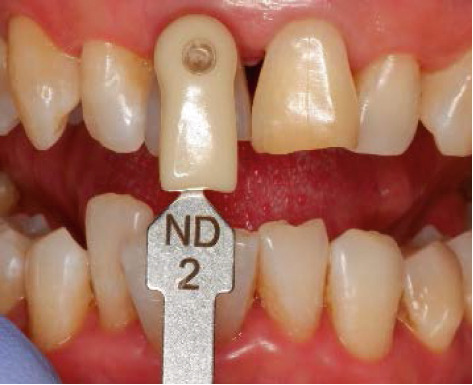
(a)

**Figure:**
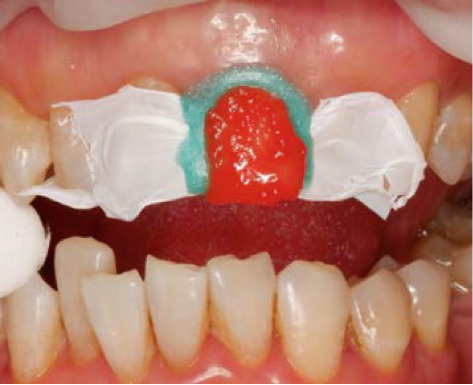
(b)

**Figure:**
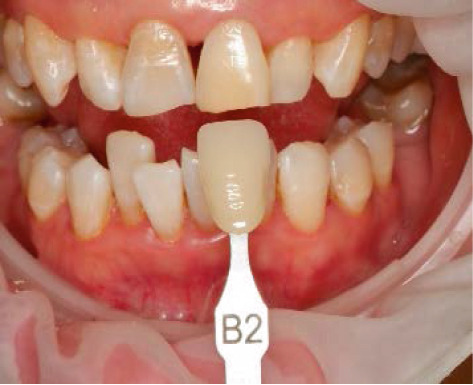
(c)

**Figure 7:**
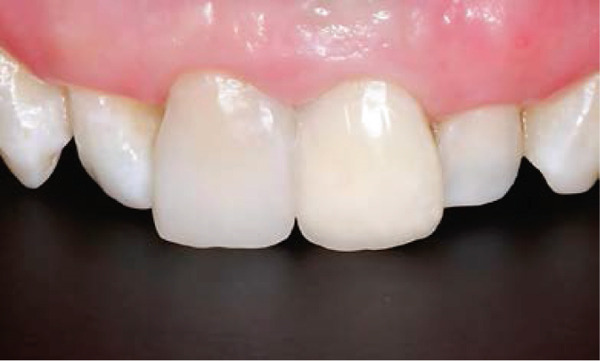
Black triangle closure using direct composite restoration.

Follow‐up was done after 1 week, the tooth remained asymptomatic without any radiographic changes, and the tooth shade previously recorded was stable at B2 shade (Ivoclar, Vivadent). After removing the temporary restoration, direct restoration with nanohybrid composite was performed to close the black triangle between the upper central incisor (Figure 7). Follow‐up was then conducted in 3, 6, 12, and 24 months.

## 3. Discussion

The outcome of dental trauma depends on the type of injury, time prior to emergency treatment, and quality of treatment. Consideration has to be given to the fact that complications of dental trauma can occur several months or even years after the injury. Delayed treatment of traumatic dental injuries (TDI) has an unfavorable effect on the prognosis of injured teeth, which can cause loss of pulp vitality, infection of the root canal system, pulp canal obliteration, crown discoloration, root resorption, bone resorption, and ankylosis [16]. Trauma between the ages of 8 and 10 years is significant because the root development of permanent teeth is often incomplete, and pulp necrosis can prevent further root development [1].

Endodontic considerations are similar in many ways in elderly patients as in younger patients, but there are many challenges due to biological, medical, physical, and cognitive limitations [17]. The reparative capacities of the pulp of older individuals will not be similar to those of young teeth. The blood supply decreases, cross‐linking between collagen fibers in the pulp decreases, and the calcium content increases with age. Cementum gets resorbed both in resorption defects and generally over the root, especially in the apical half to compensate for tooth wear during function. The rate of resorption and the number of resorption areas upsurge with age. Gingival recession exposes the cervical cementum due to environmental factors [4]. Both internal and external tooth discoloration occur in older patients. Internal discoloration is related to trauma, pulp necrosis, dental procedures, or to an increase in dentin formation with a loss of translucency. External discoloration occurs from stains and also from restorative procedures. Overall, teeth tend to discolor with time and with age [1].

In this case, periapical radiography revealed an immature tooth with Stage IV on Cvek′s classification and EIR. The thin dentinal walls increase the risk of future root fracture of the tooth under occlusal forces or iatrogenically during endodontic treatment. The root canal, which had a slightly convergent apex, gave an advantage of apical closure but had the risk of iatrogenic mishaps. Thus, in this case, hybrid technique preparation was used to accomplish the aim of mechanical preparation: remove infected soft and hard tissues, facilitate the delivery of root canal irrigants and medicaments to the apical area of the root canal system, and preserve the integrity of the root canal structure. The hybrid technique is a commonly used approach that combines the benefits of different motions and instrument sequences to produce an optimal root canal preparation outcome. This combination consists of brushing motion and balanced force motion. The advantages of brushing motion in the ProTaper files include the ability to brush laterally and selectively cut dentin on the outstroke. This brushing action helps to maintain the files in a centered position within the canal, effectively maximizing the preservation of dentin. Using balanced force motion in this sequence increases cleaning effectiveness, decreases debris extrusion, and reduces the loss of working length. Moreover, less transportation is expected due to the centric placement of the files and tactile sense when using hand files [11].

Root canal preparation was aimed at facilitating optimal disinfection and creating space for the delivery of irrigants. Besides irrigation solution, calcium hydroxide has been investigated to determine the pH changes in root dentine, and it is a powerful antibacterial agent, especially when used for the management of EIR. When calcium hydroxide is used as a root canal medicament, it releases hydroxyl ions, which diffuse through the dentinal tubules and cementum to reach the periodontal ligament. It can arrest inflammatory resorption by inhibiting the clastic cells [18].

The three‐dimensional sealing of the root canal space with a biocompatible material is the final goal of the endodontic treatment, thus preventing the passage of microorganisms, toxins, and fluids to periradicular tissues [19]. Bioceramic‐based sealers use the moisture naturally present in the dentinal tubules to commence and complete their setting reaction because they are hydrophilic and insoluble. To provide a hermetic seal, the bonding of root canal sealer to the dentine is paramount in maintaining the integrity of the seal and strengthening the root against root fracture. Calcium silicate–based sealers form a chemical link with the radicular dentin, as hydroxyapatite is produced during the setting process presenting better results considering the fracture resistance [19, 20]. Based on the study of Phukan et al., bioceramic sealer showed the highest push‐out bond strength values among the other sealers. Minimal instrumentation with a hybrid technique and bioceramic sealer improves the prognosis and helps prevent long‐term fracture risk [21].

The apical sealing ability of a root canal sealer is essential to prevent communication with periapical tissues and ensure treatment success [22]. Kharouf et al. demonstrated that bioceramic sealers effectively control bacterial growth due to their bioactive properties. CeraSeal, in particular, exhibits superior filling ability and lower solubility than other sealers, attributed to its unique chemical formulation and premixed consistency [23]. A clinical study by Spinelli et al. reported over 90% success after 36 months using a premixed bioceramic sealer with a single‐cone technique, with complete periapical healing in all cases of pulpal disease and necrosis [24]. Moreover, bioceramic sealers, such as CeraSeal, promote balanced cytokine expression (IL‐6, IL‐8, and TNF‐*α*), showing low cytotoxicity and minimal inflammatory response. These features support bioceramic sealers as advanced materials combining effective sealing, antimicrobial activity, and bioactivity for predictable long‐term endodontic outcomes [25].

Pulp hemorrhage and decomposition of pulp due to traumatic injury can cause tooth discoloration. Bleaching is a procedure that involves lightening the color of the tooth through the application of a chemical agent, which oxidizes the organic pigmentation in the tooth. In some cases, nonvital teeth can be bleached with the combination of two techniques: internal bleaching (walking bleach) and external bleaching (in office). One of the most important properties of a bleaching agent is its ability to penetrate the tooth through dentinal permeability. The deeper the penetration, the more effectively the pigments causing chromatic alteration of the dental tissue can be reversed by the oxidation reaction [26].

Bleaching can cause several side effects. One serious risk is external root resorption, which happens when bleaching agents like hydrogen peroxide pass through the tooth and damage the surrounding tissues. This can lead to inflammation and the loss of root structure, especially if heat is used, so the application of a protective barrier inside the tooth is very important. The gums should be protected during the procedure because bleaching agents can also cause chemical burns to the gums. Another problem is that leftover oxygen from bleaching can stop composite fillings from sticking properly. Using an antioxidant like sodium ascorbate can help remove this oxygen before placing a filling [1]. The discolored tooth had suffered from traumatic injury for more than 30 years, and hydrogen peroxide 35% was able to penetrate deeply into dentinal tubules and lighten the tooth when using a proper technique to prevent the side effects of the oxidizing agent.

The patient was very satisfied with the return of the color of her teeth so that she could smile brightly. There were no complaints after the root canal treatment and the color of the tooth was stable. The patient is instructed to evaluate at regular intervals to see the healing of external inflammation resorption.

## 4. Conclusion

Endodontic treatment of a necrotic immature permanent tooth due to traumatized injury in geriatric presents a significant challenge to dentists. Root canal preparation using a hybrid technique, combined with a bioceramic sealer and warm vertical compaction obturation, results in healing of the periapical lesion. Internal bleaching (walking bleach), combined with external in‐office bleaching, successfully addresses the coronal discoloration, followed by direct composite restoration achieving a favorable aesthetic outcome.

## Ethics Statement

Ethics approval is not required. Patient consent was obtained.

## Consent

The patient provided informed consent for the publication of this data.

## Conflicts of Interest

The authors declare no conflicts of interest.

## Author Contributions

Diani Prisinda: conceptualization (lead), methodology, and review. Kartika Tria Sulendra: performed the treatments, writing, and visualization. Dudi Aripin: conceptualization (supporting), review, and supervision.

## Funding

No funding was received for this manuscript.

## Data Availability

The data that support the findings of this study are available from the corresponding author upon reasonable request.

## References

[1] Gopikrishna V. , Grossman′s Endodontic Practice, 2021, 14th edition, Wolters Kluwer Health.

[2] Chotvorrarak K. , Danwittayakorn S. , Banomyong D. , and Suksaphar W. , Intraradicular Reinforcement of Traumatized Immature Anterior Teeth After MTA Apexification, Dental Traumatology. (2024) 40, no. 7, 10.1111/edt.12947. 38459664

[3] Boukpessi T. , Cottreel L. , and Galler K. M. , External Inflammatory Root Resorption in Traumatized Immature Incisors: MTA Plug or Revitalization? A Case Series, Children. (2023) 10, no. 7, 10.3390/children10071236, 37508733. PMC10377819 37508733

[4] Rajan V. V. , Jeph V. , Sharma D. , Bansal M. , and Jani M. , Endodontic Consideration in Geriatric Patients, International Journal of Applied Dental Sciences. (2022) 8, no. 2, 404–408, 10.22271/oral.2022.v8.i2f.1537.

[5] Rossi-Fedele G. and Ng Y. L. , Effectiveness of Root Canal Treatment for Vital Pulps Compared With Necrotic Pulps in the Presence or Absence of Signs of Periradicular Pathosis: A Systematic Review and Meta-Analysis, International Endodontic Journal. (2023) 56, no. S3, 370–394, 10.1111/iej.13833, 36107038. 36107038

[6] Patel B. , Endodontic Treatment, Retreatment, and Surgery: Mastering Clinical Practice, 2016, Springer, 10.1007/978-3-319-19476-9, 2-s2.0-85030615650.

[7] Patel S. , Saberi N. , Pimental T. , and Teng P. H. , Present Status and Future Directions: Root Resorption, International Endodontic Journal. (2022) 55, no. S4, 892–921, 10.1111/iej.13715, 35229320. 35229320 PMC9790676

[8] Çalışkan M. K. and Kaval M. E. , Endodontic Management of Immature Teeth With Spontaneous Apical Closure and Periapical Lesions: Case Series and Review of the Literature, Dental Traumatology. (2015) 31, no. 4, 324–327, 10.1111/edt.12164, 2-s2.0-84934808307, 25731672. 25731672

[9] Harlamb S. C. , Management of Incompletely Developed Teeth Requiring Root Canal Treatment, Australian Dental Journal. (2016) 61, no. S1, 95–106, 10.1111/adj.12401, 2-s2.0-84959888497, 26923451. 26923451

[10] Rodrigues R. C. V. , Zandi H. , Kristoffersen A. K. , Enersen M. , Mdala I. , Ørstavik D. , Rôças I. N. , and Siqueira J. F.Jr., Influence of the Apical Preparation Size and the Irrigant Type on Bacterial Reduction in Root Canal–Treated Teeth With Apical Periodontitis, Journal of Endodontics. (2017) 43, no. 7, 1058–1063, 10.1016/j.joen.2017.02.004, 2-s2.0-85018782098, 28483164. 28483164

[11] Bolourchi I. and Pourmousavi L. , A Novel Hybrid Hand Instrumentation Technique for Root Canal Preparation, Iranian Endodontic Journal. (2018) 13, no. 4, 461–468, 10.22037/iej.v13i4.19179, 2-s2.0-85056592627, 36883023. 36883023 PMC9985688

[12] Eid D. , Medioni E. , De-Deus G. , Khalil I. , Naaman A. , and Zogheib C. , Impact of Warm Vertical Compaction on the Sealing Ability of Calcium Silicate-Based Sealers: A Confocal Microscopic Evaluation, Materials. (2021) 14, no. 2, 10.3390/ma14020372, 33466619. PMC7828729 33466619

[13] Berlin-Broner Y. , Al Bawaliz L. , and Levin L. , Implications of Post-Traumatic Treatment of Immature Maxillary Incisors, International Dental Journal. (2023) 73, no. 3, 337–345, 10.1016/j.identj.2023.01.005, 36804746. 36804746 PMC10213731

[14] Amer M. , Intracoronal Tooth Bleaching – A Review and Treatment Guidelines, Australian Dental Journal. (2023) 68, no. S1, S141–S152, 10.1111/adj.13000. 37975331

[15] Knezevic N. , Obradovic M. , Dolic O. , Veselinovic V. , Kojic Z. , Josipovic R. , and Arapovic-Savic M. , Clinical Testing of Walking Bleach, In-Office, and Combined Bleaching of Endodontically Treated Teeth, Medicina (Lithuania). (2023) 59, no. 1, 10.3390/medicina59010018, 36676642. PMC9865317 36676642

[16] Chaudhary S. , Singh H. , Gharti A. , and Adhikari B. , Evaluation of Clinical and Radiographic Findings Among Patients With Traumatic Dental Injuries Seeking Delayed Treatment, International Journal of Dentistry. (2021) 2021, 9549508, 10.1155/2021/9549508, 34471410. 34471410 PMC8405333

[17] Alrahabi M. K. , Root Canal Treatment in Elderly Patients: A Review and Clinical Considerations, Saudi Medical Journal. (2019) 40, no. 3, 217–223, 10.15537/smj.2019.3.23769, 2-s2.0-85062419483, 30834415. 30834415 PMC6468204

[18] Abbott P. V. , Prevention and Management of External Inflammatory Resorption Following Trauma to Teeth, Australian Dental Journal. (2016) 61, no. S1, 82–94, 10.1111/adj.12400, 2-s2.0-84959897048, 26923450. 26923450

[19] Falakaloğlu S. and Gündoğar M. , Evaluation of the Push Out of Bond Strength of Different Bioceramic Root Canal Sealers With Different Obturation Techniques, Giornale Italiano di Endodonzia. (2022) 36, no. 1, 151–157.

[20] Lichaa R. , Deeb G. , Mhanna R. , and Zogheib C. , Comparison of Fracture Resistance Between Single-Cone and Warm Vertical Compaction Technique Using Bio-C Sealer in Mandibular Incisors: An In Vitro Study, Journal of Contemporary Dental Practice. (2022) 23, no. 2, 143–148, 10.5005/jp-journals-10024-3311, 35748441. 35748441

[21] Phukan A. H. , Mathur S. , Sandhu M. , and Sachdev V. , The Effect of Different Root Canal Sealers on the Fracture Resistance of Endodontically Treated teeth-in Vitro Study, Dental Research Journal. (2017) 14, no. 6, 10.4103/1735-3327.218558, 2-s2.0-85034955267. PMC5713061 29238376

[22] Rekha R. , Kavitha R. , Venkitachalam R. , Singh V. P. , Deepthy S. , and Krishnan V. , Comparison of the Sealing Ability of Bioceramic Sealer Against Epoxy Resin Based Sealer: A Systematic Review & Meta-Analysis, Journal of Oral Biology and Craniofacial Research. (2023) 13, no. 1, 28–35, 10.1016/j.jobcr.2022.10.006, 36345495. 36345495 PMC9636474

[23] Kharouf N. , Arntz Y. , Eid A. , Zghal J. , Sauro S. , Haikel Y. , and Mancino D. , Physicochemical and Antibacterial Properties of Novel, Premixed Calcium Silicate-Based Sealer Compared to Powder–Liquid Bioceramic Sealer, Journal of Clinical Medicine. (2020) 9, no. 10, 10.3390/jcm9103096. PMC7600315 32992852

[24] Spinelli A. , Zamparini F. , Lenzi J. , Gandolfi M. G. , and Prati C. , Three-Year Clinical Outcome of Root Canal Treatment Using a Single-Cone Technique and CeraSeal Premixed Bioceramic Sealer: A Prospective Cohort Study, European Endodontic Journal. (2024) 9, no. 4, 383–393, 10.14744/eej.2024.75537, 39704630. 39704630 PMC11685523

[25] Gaafar S. S. , El Mekkawi A. R. O. , Farag R. A. , Gadelmawla M. H. A. , Hussein A. M. H. M. , Sayed M. , and El-Sherbiny R. , Comparative Analysis of the Inflammatory Response of Human Gingival Fibroblasts to NeoSEALER Flo and CeraSeal Bioceramic Sealers: An In Vitro Study, BMC Oral Health. (2025) 25, no. 1, 10.1186/s12903-025-05692-1, 40098184. PMC11917106 40098184

[26] Rokaya M. E. , Evaluation of Extraradicular Diffusion of Hydrogen Peroxide During Intracoronal Bleaching Using Different Bleaching Agents, International Journal of Dentistry. (2015) 2015, 493795, 10.1155/2015/493795, 2-s2.0-84938124760, 26257782. 26257782 PMC4516840

